# Supplementary material for: Borrowing of information across patient subgroups in a basket trial based on distributional discrepancy
Source: Biostatistics. 2020 May 7;23(1):120–35. doi: 10.1093/biostatistics/kxaa019 (PMC8759447; doi:10.1093/biostatistics/kxaa019)
Supplement: kxaa019_Supplementary_Data [file kxaa019_supplementary_data.pdf]

# **Web-based Supplementary Materials for**: Borrowing of information across patient subgroups in a basket trial based on distributional discrepancy

**by Haiyan Zheng, James M.S. Wason**

### A. TRANSFORMATION OF PAIRWISE DISTANCES INTO PROBABILITY WEIGHTS

In the main manuscript, we used Equation (3.11) to transform the pairwise Hellinger distances into probability weights, which contribute to combining the commensurate predictive priors specified based on subtrial data $\boldsymbol{x}_k$ complementary to subtrial $k^\star$ of our analysis interest. In this section, we present the property of this transformation analytically. How different values of $s_0$ may impact the trial operating characteristics is illustrated numerically with simulation results in Section C.

> **Property 1**: Given a vector of equal pairwise $d_{kk^\star}$, equal weights $p_{kk^\star} = \frac{1}{K-1}$ will be obtained for any $s_0 > 0$.

> **Property 2**: A large value of $s_0 \gg 1 \geq d_{kk^\star}$ yields about equal weights $p_{kk^\star} \to \frac{1}{K-1}$ irrespective of the magnitude of differences between $d_{kk^\star}$.

**Proof**: Suppose all the pairwise $d_{kk^\star}$ are identical to $0 \leq d \leq 1$. Following Equation (3.11), we have

$$p_{kk^\star} = \frac{\exp(-d/s_0)}{\underbrace{\exp(-d/s_0) + \cdots + \exp(-d/s_0)}_{(K-1)\text{ terms}}} = \frac{1}{K-1}.$$

We constrain $s_0 > 0$ so as to have a decreasing function of the distance measure. On the other hand, when $s_0 \gg 1 \geq d_{kk^\star}$, $\exp(-d_{kk^\star}/s_0) \to \exp(0) = 1$. Therefore, $p_{kk^\star} \to \frac{1}{K-1}$.

> **Proposition**: The difference between weights $p_{kk^\star}$ transformed from $0 \leq d_{kk^\star} \leq 1$ decreases, as the value of $s_0$ increases.

**Proof**: Let $d_{tk^\star}$ and $d_{jk^\star}$ be any two pairwise Hellinger distances from the $k^\star$-th column of the $K \times K$ distance matrix, and we further suppose that $d_{tk^\star} \leq d_{jk^\star}$. Let $p_{tk^\star}$ and $p_{jk^\star}$ be the weights obtained following Equation (3.11), respectively, in the presence of other pairwise distances to $\theta_{k^\star}$. Denoting the difference by $\Delta_p = p_{tk^\star} - p_{jk^\star}$, we derive the derivative of $\Delta_p$ with respect to $s_0$ to illustrate how $\Delta_p$ changes (increase or decrease), as $s_0$ increases.

$$\begin{aligned}
\Delta_p &= p_{tk^\star} - p_{jk^\star} \\
&= \frac{\exp(-d_{tk^\star}/s_0)}{\sum_k \exp(-d_{kk^\star}/s_0)} - \frac{\exp(-d_{jk^\star}/s_0)}{\sum_k \exp(-d_{kk^\star}/s_0)} \\
&= \frac{\exp(-d_{tk^\star}/s_0) - \exp(-d_{jk^\star}/s_0)}{\sum_k \exp(-d_{kk^\star}/s_0)}
\end{aligned} \tag{1}$$

Let $f = \exp(-d_{tk^\star}/s_0) - \exp(-d_{jk^\star}/s_0)$ and $g = \sum_k \exp(-d_{kk^\star}/s_0)$. Following the *quotient rule*, we know that $\Delta'_p = \frac{f'g - fg'}{g^2}$. The interest is to learn the rate of change of $\Delta_p$ changes as the value of $s_0$ varies, through the derivative. In the following, we obtain $f'$ and $g'$, respectively.

$$
\begin{aligned}
f' &= \left\{\exp\left(-\frac{d_{tk^\star}}{s_0}\right)\right\}' - \left\{\exp\left(-\frac{d_{jk^\star}}{s_0}\right)\right\}' \\
&= \frac{1}{s_0^2} d_{tk^\star} \exp\left(-\frac{d_{tk^\star}}{s_0}\right) - \frac{1}{s_0^2} d_{jk^\star} \exp\left(-\frac{d_{jk^\star}}{s_0}\right),
\end{aligned} \tag{2}
$$

and

$$
\begin{aligned}
g' &= \left[\sum \exp\left(-\frac{d_{kk^\star}}{s_0}\right)\right]' \\
&= \left[\exp\left(-\frac{d_{1k^\star}}{s_0}\right) + \exp\left(-\frac{d_{2k^\star}}{s_0}\right) + \cdots + \exp\left(-\frac{d_{Kk^\star}}{s_0}\right)\right]' \\
&= \exp\left(-\frac{d_{kk^\star}}{s_0}\right) \cdot (-d_{1k^\star}) \cdot (-1) s_0^{-2} + \cdots + \exp\left(-\frac{d_{Kk^\star}}{s_0}\right) \cdot (-d_{Kk^\star}) \cdot (-1) s_0^{-2} \\
&= s_0^{-2} \left[d_{1k^\star} \exp\left(-\frac{d_{1k^\star}}{s_0}\right) + \cdots + d_{Kk^\star} \exp\left(-\frac{d_{Kk^\star}}{s_0}\right)\right].
\end{aligned} \tag{3}
$$

Thus,

$$
\begin{aligned}
f'g - fg' &= \left[\frac{1}{s_0^2} d_{tk^\star} \exp\left(-\frac{d_{tk^\star}}{s_0}\right) - \frac{1}{s_0^2} d_{jk^\star} \exp\left(-\frac{d_{jk^\star}}{s_0}\right)\right] \left[\exp\left(-\frac{d_{1k^\star}}{s_0}\right) + \cdots + \exp\left(-\frac{d_{Kk^\star}}{s_0}\right)\right] \\
&\quad - \left[\frac{1}{s_0^2} \exp\left(-\frac{d_{tk^\star}}{s_0}\right) - \frac{1}{s_0^2} \exp\left(-\frac{d_{jk^\star}}{s_0}\right)\right] \left[d_{1k^\star} \exp\left(-\frac{d_{1k^\star}}{s_0}\right) + \cdots + d_{Kk^\star} \exp\left(-\frac{d_{Kk^\star}}{s_0}\right)\right] \\
&\leq \left[\frac{1}{s_0^2} d_{tk^\star} \exp\left(-\frac{d_{tk^\star}}{s_0}\right) - \frac{1}{s_0^2} d_{jk^\star} \exp\left(-\frac{d_{jk^\star}}{s_0}\right)\right] \left[\exp\left(-\frac{d_{1k^\star}}{s_0}\right) + \cdots + \exp\left(-\frac{d_{Kk^\star}}{s_0}\right)\right] \\
&\quad - \left[\frac{1}{s_0^2} \exp\left(-\frac{d_{tk^\star}}{s_0}\right) - \frac{1}{s_0^2} \exp\left(-\frac{d_{jk^\star}}{s_0}\right)\right] \left[\exp\left(-\frac{d_{1k^\star}}{s_0}\right) + \cdots + \exp\left(-\frac{d_{Kk^\star}}{s_0}\right)\right] \\
&= \frac{1}{s_0^2} \left[\exp\left(-\frac{d_{1k^\star}}{s_0}\right) + \cdots + \exp\left(-\frac{d_{Kk^\star}}{s_0}\right)\right] \left[\exp\left(-\frac{d_{tk^\star}}{s_0}\right)(d_{tk^\star} - 1) - \exp\left(-\frac{d_{jk^\star}}{s_0}\right)(d_{jk^\star} - 1)\right] \leq 0
\end{aligned} \tag{4}
$$

The last step of the inequality follows, because $h(d) = \exp(-\frac{d}{s})(d-1)$ is monotonically increasing as $d$ increases, at a given value of $s$.

Therefore, $\Delta_p' = \frac{f'g - fg'}{g^2} \leq 0$, which means $\Delta_p$ is decreasing for all $s_0$ in the interior of $\mathbb{S} = (0, \infty)$. It suggests that a smaller value of $s_0$ discerns the differences between $d_{kk^\star}$ more sensitively. This Proposition can also be linked to Property 2 that we argue against setting $s_0 \gg 1 \geq d_{kk^\star}$.

## B. SPECIFYING THE SPIKE-AND-SLAB PRIOR

We place a spike-and-slab prior on the precision factor of the predictive prior, $\nu$. Data from an incommensurate subtrial are down-weighted through the 'slab' prior Unif($\mathcal{B}_1, \mathcal{B}_2$), from which $\frac{1}{\nu}$ has an inverse uniform distribution with the probability density function as $g_k(x) = (\mathcal{B}_2 - \mathcal{B}_1)^{-1} x^{-2}$ and the support on $[\mathcal{B}_2^{-1}, \mathcal{B}_1^{-1}]$. The point-to-point borrowing of information from a specific subtrial for commensurability is through the point-mass prior, $\mathcal{S}$, so-called the 'spike'.

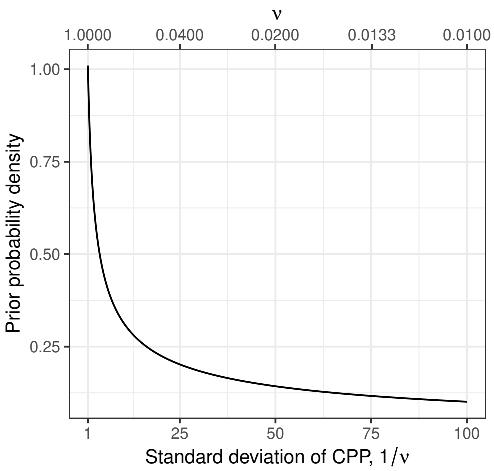


Figure S1: Prior pobability density of the standard deviation of a commensurate predictive prior (CPP) under the slab prior for down-weighting.

Following the stipulation (3.3) of the main manuscript, information on the treatment effect in subtrial $k$ is represented into a predictive distribution, with the normal precision $\nu_{kk^\star}^2$ which determines the degree of borrowing. In the simulation study, we set $\mathcal{B}_1 = 0.01$, $\mathcal{B}_2 = 1$ and $\mathcal{S} = 100$ to define the spike-and-slab for each $\nu_{kk^\star}$ for the strong down-weighting or borrowing of information in the extreme cases of prior probability $w_{kk^\star} = 1$ or 0, respectively. Relating this specification of slab-and-spike prior to the parameter configurations of generating trial data, where the standard deviation (SD) for $\theta_k$ is expected to be smaller than 0.4 (we set the SD of patient responses $y_{ik}$ as $\sigma = 0.4$). Figure S1 visualises the prior probability density distribution of the 'slab' prior used for simulations, from which we know with probability of 96.0%, the SD of the commensurate predictive prior $1/\nu_{kk^\star} \in [1, 25]$, and 98.0% that $1/\nu_{kk^\star} \in [1, 50]$. Even when $1/\nu_{kk^\star} = 1$ at the bound, this is sufficient to substantially discount the information from subtrial $k$. The 'spike' prior was specified so that $\boldsymbol{x}_k$ can be *completely pooled* into subtrial $k^\star$ for perfect data consistency, which can be suggested by a $d_{kk^\star} = 0$. In such situations, the proposed methodology regards patients of the two subtrials as exchangeable irrespective of the subgroup label.

## C. ADDITIONAL SIMULATION RESULTS

In this section, we report and interpret additional simulation results for comparing the proposed methodology with several alternative Bayesian models.

Figure S2 shows the median width of the 95% posterior credible intervals (CIs) for $\theta_k$ using bars of different heights, together with the 10th and 90th percentiles using endpoints of the error bars. This help illustrate how precise the Bayesian models can give the posterior estimates, in addition to the bias and MSE comparisons in Section 4 (Figure 1) of the main manuscript. As we can see, the approach of no borrowing leads to the widest posterior CIs across all scenarios. In contrast, the proposed approach (borrowing based on distributional discrepancy) has the narrowest posterior CIs, when there exist at least one subtrial with similar treatment effect. In scenarios 5 and 6, where data are consistent across subtrials, the proposed approach outperforms the other Bayesian models by producing the narrowest posterior CIs. Looking at, for example, subtrials 3 and 4 in scenario 1 that have relatively moderate treatment effect in a basket trial, the proposed approach leads to posterior estimates with the CI widths comparable to those of the standard HM or EXNEX,

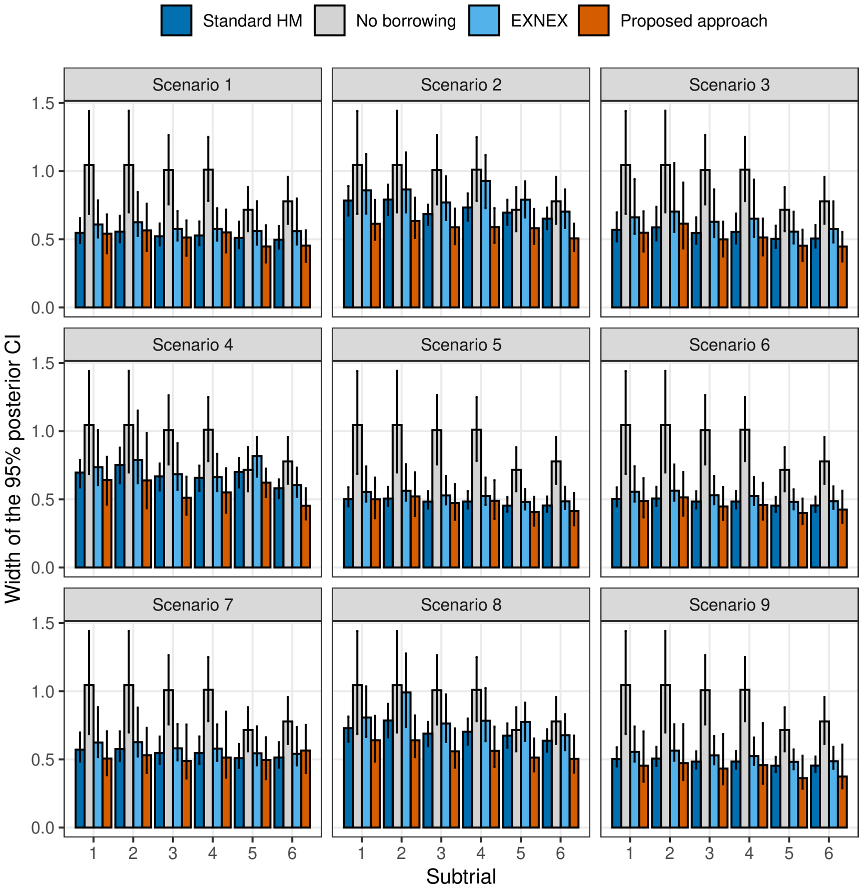


Figure S2: A bar chart for the width of the credible interval, bounded by the 2th and 97.5th percentiles, of the posterior distribution for $\theta_k$, $k = 1, \ldots, 6$ estimated using different Bayesian analysis models.

but much wider error bars. This suggests that our approach using a discrepancy measure is more sensitive to the disparate treatment effect on patient subgroups demonstrated by trial data. By identifying subtrials with similar treatment effect, it can ensure the inference for a subtrial with an extreme treatment effect with information leveraged from subtrials with the most commensurate treatment effect or no borrowing at all. Whereas, the alternative approaches of borrowing (such as the standard HM) tend to shrink the subgroup-specific estimates towards the overall population mean, which is often close to the medium effect size as an average across all subtrials.

In Section A, we demonstrated two essential properties of the transformation of the pairwise Hellinger distances into weights to identify the most commensurate subtrial(s). We now present numerically how the proposed methodology differentiates the degree of borrowing from other subtrials, which may have either commensurate or incommensurate treatment effects. Specifically, it is desirable to allocate the largest weight(s) to the most consistent complementary subtrial(s) in a scenario of heterogeneous treatment effects, but nearly equal weights in a scenario of subtrials with consistent treatment effects.

Based on 5000 simulation replicates, Figure S3 reports the weight allocation to the $(K-1)$ commensurate predictive priors, as a function of the computed pairwise commensurability, for scenarios 4 (some subtrials are more commensurate between themselves than with others) and 5 (equally commensurate between all). The subfigures show our approach can correctly identify the most consistent subtrials. Referring to subtrial 5 with the lowest treatment effect of subfigure

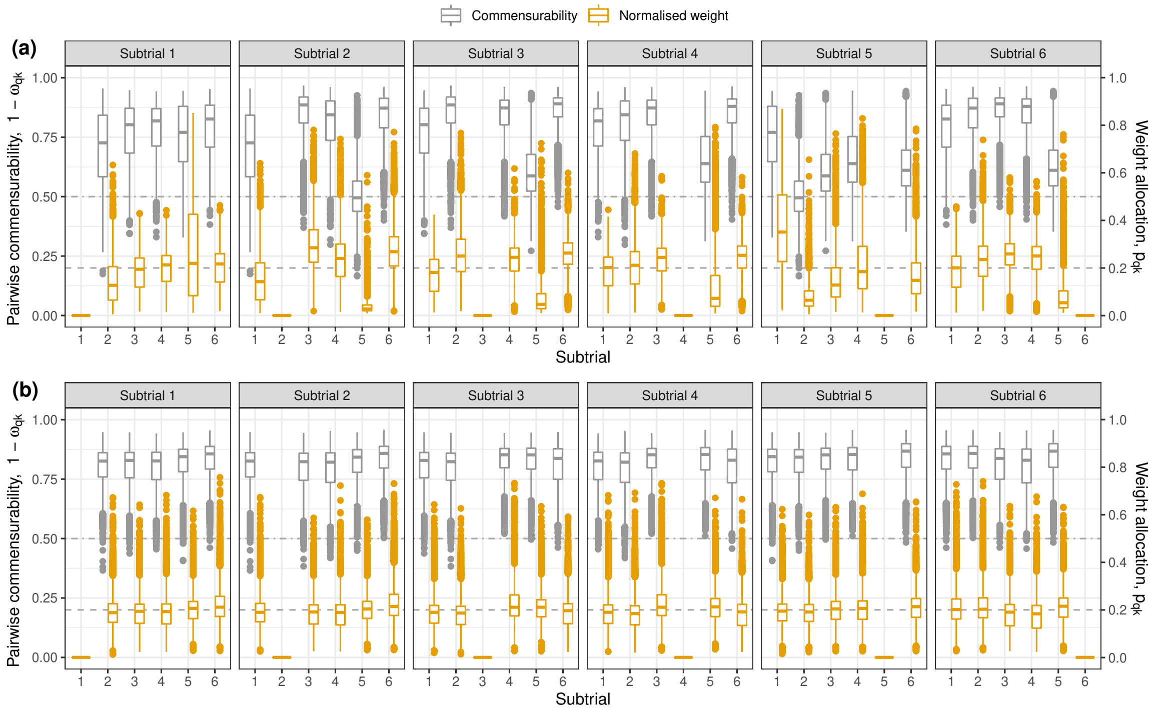


Figure S3: Boxplots of the weight allocation to a specific subtrial ($q \neq k$) for leveraging information into the subtrial $k$ of current analysis interest. Subfigures (a) and (b) visualise the simulation results of scenarios 4 and 5, repsectively. The dashed horizontal lines indicate the level of commensurability as 0.5, and the weight of 0.2 which is the case when all subtrials are equally commensurate.

(a), for example, we read the largest weight is allocated to subtrial 1 and the smallest to subtrial 2, with the medians as 0.351 and 0.06, respectively. Subfigure (b) shows that equal weights are allocated to the other subtrials in a case of all subtrials with the same treatment effect. The median weights are 0.2 for the other subtrials respectively, as is illustrated in Section A. The interquartile range of each boxplot, for pairwise commensurability and correspondingly the weight, tend to be large for subtrials that have a medium size of treatment effect. For example, within the same plot for subtrials 2 – 6 of subfigure (a), the interquartile range of the boxplot of subtrial 1 is larger than any other subtrials to the same target subtrial. Fitting a linear regression model to the simulated basket trial data, $\theta_1$ may be estimated as larger (smaller) than the true $\theta_1$ and regarded to be more commensurate to subtrials with a high (low) treatment effect, such as subtrial 2 (5).

We move onto the exploration of how the specification of $s_0$ would impact the sensitivity for identifying the most commensurate subtrial(s). Figure S4 presents boxplots of the weights, which are transformed from the same pairwise Hellinger distances, to reflect the relative importance of other subtrials in scenario 4, where we have set $s_0 = 0.15$ (used for the main simulation study), 0.25, 0.35 and 0.45. Taking the plot for Subtrial 5 in Figure S4 as an example, we observe that as $s_0$ increases, the weights allocated to other subtrials (despite the different magnitudes of incommensurability) converge to the value of 0.2. When nearly equal weights are yielded, it means that all the other subtrials are regarded as equally (in)commensurate. Robust inferences can still be achieved, as the individual point-to-point commensurate priors are determined based on the computed pairwise Hellinger distance for appropriate amount of borrowing. But setting a large value of $s_0$ leads the proposed methodology to lose the ability of identifying the commensurate subtrials sensitively. As was demonstrated in Section A, we argue against a $s_0 \gg 1 \geq d_{kk^\star}$ which would result in about equal weights for the other subtrials even if the treatment effects may vary.

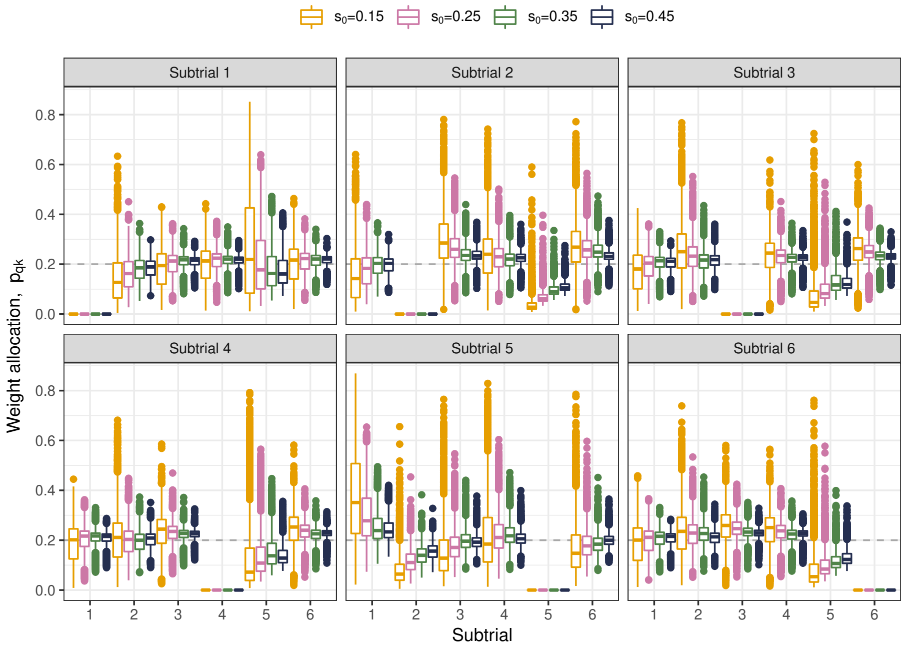


Figure S4: Boxplots of the weight allocation to complementary subtrials, transformed from the pairwise Hellinger distance setting $s_0 = 0.15, 0.25, 0.35, 0.45$ in scenario 4.

Finally, we note that the *Go* or *No-go* decision made for each subtrial depends on the interval probability computed accroding to the criterion defined in Section 2 of the main manuscript. The operating characteristics, i.e., analogues of the statistical power and type I error rate, vary relying on the choices of the threshold, $\delta_U$, and the level $\zeta$. In Section 4 of the main manuscript, we have reported a subset of the simulation results, setting $\delta_U = 0.25$ and $\zeta = 0.975$. In Figure S5 and Table S1 below, we present the complementary simulation results of operating characteristics produced from $\delta_U = 0.30$ and $\zeta = 0.975$.

Looking across Figure 2 ($\delta_U = 0.25$) of the main manuscript and Figure S5 ($\delta_U = 0.35$), we observe that the Bayesian models yield lower power in general given a larger threshold $\delta_U$. The magnitude of decrease by different models may not be the same, particularly for the approaches of borrowing, where the power curve is a function of both the treatment effect and the information leveraged from other subtrials. In particular, the stipulation of the half-normal priors and the 'spike' prior determines the maximum amount of information that can be leveraged. When the threshold $\delta_U$ is levelled up, it becomes more difficult to make a *Go* for a subtrial with $\theta_k = 0$. The analogue of type I error rate recorded in Table S1 is therefore smaller than that is in Table 2 of the main manuscript.

## D. SOME CONSIDERATIONS ON THE SUBTRIAL SAMPLE SIZE

In the main manuscript, we evaluated the proposed Bayesian method in the simulation study for analysing basket trials with unequal subtrial sample sizes. Nevertheless, the subtrial sample sizes, ranging from 10 to 20, do not vary by much. We note there are scenarios when a basket trial would be planned to include rare disease subgroup(s), in which a standard phase II trial may be unrealistic to be undertaken in light of the scant sample size. It would therefore be interesting to see how the proposed method works in those cases, as pointed out by a reviewer. We are

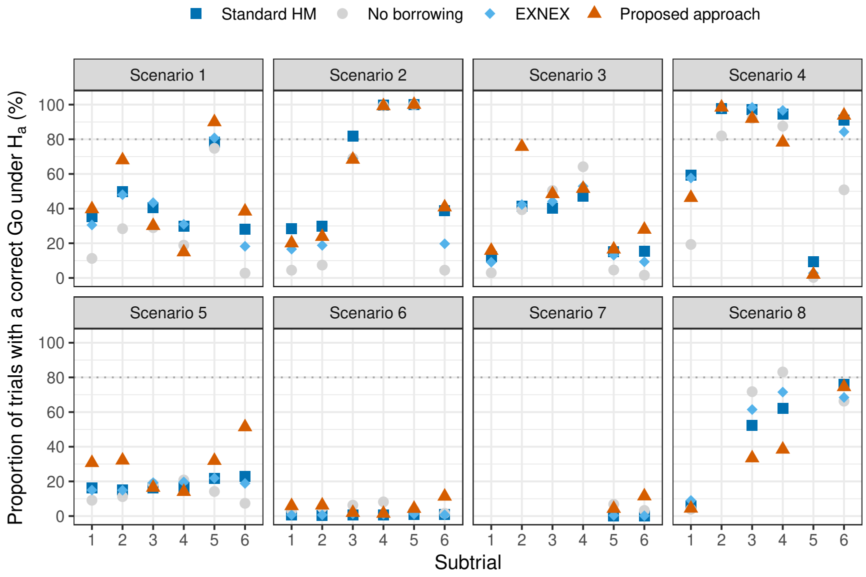


Figure S5: Comparison of the Bayesian analysis models with respect to the analogue of statistical power: null hypothesis is correctly rejected in the presence of a treatment effect per subtrial, setting $\delta_U = 0.30$ and $\zeta = 0.975$.

Table S1: Comparison of the Bayesian analysis models with respect to the analogue of type I error rate: null hypothesis is erroneously rejected under scenarios of any $\theta_k = 0$, setting $\delta_U = 0.30$ and $\zeta = 0.975$.

| | | Subtrial | | | | | | |
|---|---|---|---|---|---|---|---|---|
| | | 1 | 2 | 3 | 4 | 5 | 6 | **Overall** |
| **Scenario 7** | Standard HM | 0.0000 | 0.0000 | 0.0000 | 0.0000 | - | - | 0.0000 |
| | No borrowing | 0.0025 | 0.0052 | 0.0035 | 0.0063 | - | - | 0.0175 |
| | EXNEX | 0.0000 | 0.0000 | 0.0000 | 0.0000 | - | - | 0.0000 |
| | Proposed approach | 0.0033 | 0.0055 | 0.0001 | 0.0000 | - | - | 0.0089 |
| **Scenario 8** | Standard HM | - | 0.0058 | - | - | 0.0026 | - | 0.0084 |
| | No borrowing | - | 0.0052 | - | - | 0.0004 | - | 0.0056 |
| | EXNEX | - | 0.0100 | - | - | 0.0022 | - | 0.0122 |
| | Proposed approach | - | 0.0032 | - | - | 0.0000 | - | 0.0032 |
| **Scenario 9** | Standard HM | 0.0000 | 0.0000 | 0.0000 | 0.0000 | 0.0000 | 0.0000 | 0.0000 |
| | No borrowing | 0.0025 | 0.0052 | 0.0035 | 0.0063 | 0.0004 | 0.0004 | 0.0182 |
| | EXNEX | 0.0000 | 0.0001 | 0.0000 | 0.0000 | 0.0000 | 0.0000 | 0.0001 |
| | Proposed approach | 0.0009 | 0.0017 | 0.0000 | 0.0000 | 0.0000 | 0.0008 | 0.0034 |

* **Overall**: the proportion of trials with erroneous *Go* decision for at least one subtrial.

particularly concerned about whether effective borrowing of information could enhance inference for a small subtrial, when the sufficient statistics (i.e., mean and variance) of the subtrial data are nearly identical apart from the fact that $n_q \ll n_k$, with $q \neq k$. For simplicity, in the hypothetical data examples that we will present and interpret below, we constrain that the basket trials involve two subgroups only, thus $K = 2$.

We simulate the hypothetical basket trial data setting the 'true' parameter value for $\theta_1 = \theta_2 = 0.8$ and following the settings in Section 4.1 of the main manuscript, under two realistic scenarios where $n_1 = n_2 = 20$ and where $n_1 = 20, n_2 = 200$. In particular, the trial structure and the parameters other than $\theta_k$ for generating and analysing the basket trial data remain unchanged.

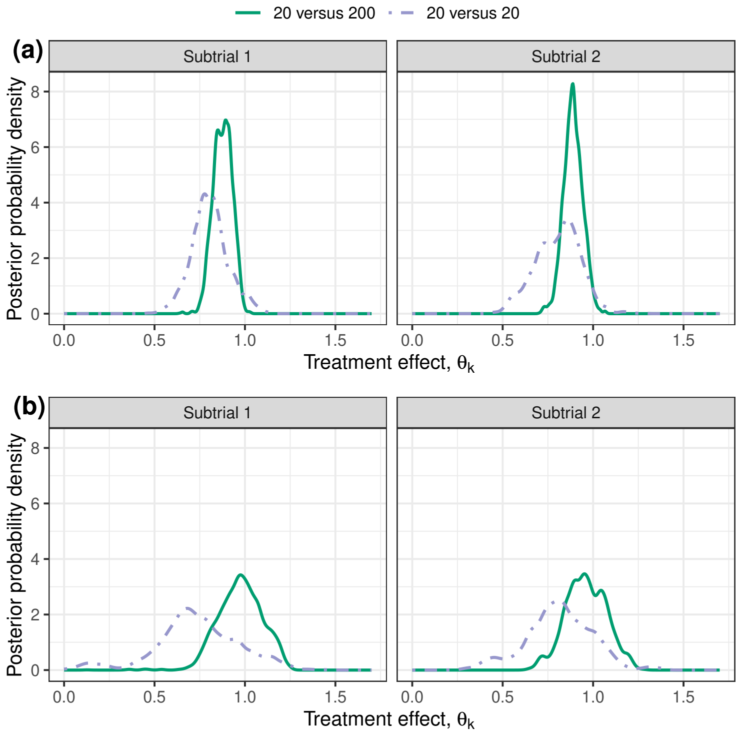


Figure S6: Posterior pobability density of the treatment effect $\theta_k$, with information leveraged from a complementary subtrial with a similar or different sample size. Subfigures (a) and (b) correspond to the analysis results of data simulated by setting the inter-patient standard deviation $\sigma = 0.4$ and 0.8, respectively.

We present two sets of hypothetical data examples below. In the first set with relatively small inter-patient variability ($\sigma = 0.4$), the operational posteriors are approximately $N(0.808, 0.12^2)$ and $N(0.804, 0.11^2)$ in the scenario of $n_1 = n_2 = 20$, which suggests the subtrial data as highly commensurate. The strong borrowing of information is permitted following our methodology, as evidenced by the Hellinger distance eventually assessed as $d_H(\pi_{\theta_1}, \pi_{\theta_2}) = 0.05$ for the posteriors leveraging complementary subtrial data. For comparison, in the scenario where $n_1 = 20, n_2 = 200$, the operational posteriors are approximately $N(0.762, 0.19^2)$ and $N(0.863, 0.05^2)$, respectively. The Hellinger distance between the posteriors based on all subtrial data is 0.08, which is about the same level of commensurability as that of the scenario of equal subtrial sample size. In the second set of data examples with a larger inter-patient variability ($\sigma = 0.8$), the operational posteriors are approximately $N(0.484, 0.27^2)$ and $N(0.463, 0.28^2)$ in the equal subtrial sample size scenario, and $N(1.277, 0.55^2)$ and $N(0.884, 0.15^2)$ in the scenario of $n_1 = 20, n_2 = 200$. The Hellinger distances between the posteriors based on all subtrial data are both 0.16 for the two sample size scenarios.

Figure S6 visualises the posterior density curves of $\theta_k$ with information leveraged from the complementary subtrial based on the proposed method. Subfigure (a) corresponds to the first hypothetical dataset (with $\sigma = 0.4$) and subfigure (b) to the second (with $\sigma = 0.8$). Sample size scenarios are distinguished by the colour and line type of the pdf curves: gray and dashed for the scenario $n_1 = n_2 = 20$, while green and solid for that of $n_1 = 20, n_2 = 200$.

Here, we would like to draw the reader's attention to the plots labelled 'Subtrial 1' within both subfigures (a) and (b), as the interest centres around the informativeness of $\theta_1$ (the treatment effect in the smaller subtrial) with data leveraged from subtrial 2 (particularly in the scenario of $n_2 = 200$). As reported, the Hellinger distances computed in both scenarios are comparable and

both suggesting high commensurability. How informative the posterior of $\theta_1$ could be depends on (i) the data exclusively from subtrial 1, and (ii) the complementary subtrial data together with the Hellinger distance. Comparing the gray with the green curves (across sample size scenarios) in the Subtrial 1 plots, we observe that incorporating the complementary data with $n_2 = 200$ increases the informativeness of the posterior for $\theta_1$ substantially. It drags the curve shape (height and dispersion) towards that of the green curve shape in the plot labelled 'Subtrial 2' which is largely determined by the subtrial data with $n_2 = 200$ in the same subfigure.

These data examples suggest that the proposed approach can accommodate circumstances when the subtrial sample sizes of a basket trial are imbalanced. Since the Hellinger distance guides the magnitude of borrowing through the distribution parameters (estimated mean and variance of the operational posterior), the complementary subtrial data, regardless of the actual sample size, can be leveraged properly in the considered setup (where the sample size of the small subtrial is not that extreme). However, this area deserves further research for more extreme scenarios with $n_1 \ll n_2$. Additional complexity would arise if the inter-patient variability is different across subtrials. It is beyond the scope of the present paper, but we envisage it could be significant, and also relevant to future basket trials which may adopt asynchronous recruitment. For example, by the time a new subtrial is open to enroll patients, some complementary subtrials have already accrued a good amount of information.
